# Improved maize technology adoption and its intensity among male- and female-headed households in Dawuro zone, Southwestern Ethiopia

**DOI:** 10.1080/09718524.2022.2140382

**Published:** 2022-12-28

**Authors:** Girma Gezimu Gebre, Yuichiro Amekawa, Dil Bahadur Rahut

**Affiliations:** a Faculty of Gender, Environment and Development Studies, College of Agriculture, Hawassa University, Hawassa, Ethiopia;; b JSPS Postdoctoral Research Fellow at Ritsumeikan University, Kyoto, Japan;; c College of International Relations, Ritsumeikan University, Kyoto, Japan;; d Asian Development Bank Institute, Tokyo, Japan

**Keywords:** Gender difference, male and female-headed households, technology adoption, adoption intensity, double-hurdle model, maize, Dawuro zone, Ethiopia

## Abstract

Both male- and female-headed farm households grow maize in Ethiopia. However, little is known about the difference between male- and female-headed households in the adoption of high-yielding technologies for maize. This study examines the difference between male- and female-headed households in their decision to adopt and the intensity of adoption of improved maize technologies in Dawuro zone, Southwestern Ethiopia. The study uses primary data collected in 2018 from 560 maize-producing households in Dawuro zone, Ethiopia. As the female-headed households are not homogenous, this study separately assessed gender differences in improved maize technology adoption between *de facto* female-headed households and *de jure* female-headed households. The results show that the intensity of adoption of improved maize varieties (IMVs) and chemical fertilizers on plots managed by male-headed households is statistically different from those managed by female-headed households. The intensity of adoption of IMVs and chemical fertilizers by female-headed household is lower compared to male-headed households. As economic status is a key driver of the adoption of IMVs and fertilizer application, it is recommended that the policies and programs that aim at developing and disseminating quality maize seeds and fertilizers in Dawuro zone should support economically poor female-headed households, particularly, *de jure* female heads.

## Introduction

Maize is the most widely grown crop in Ethiopia, and it accounts for the largest share of the total crop production. Between 2006 and 2017, the national average yield was about 2.6 t/ha (Central Statistical Agency [CSA], 2015, 2017), which is higher than the sub-Saharan African average of 1.8 t/ha (FAO, 2018). However, if farmers would adopt improved technologies, the current maize yield could be doubled (CSA, 2017). Both male- and female-headed farm households grow maize in Ethiopia. However, little is known about gender differences in the adoption of high-yielding technologies for maize.

An extensive body of literature has examined the determinants that affect farmers’ adoption of agricultural technologies (Doss et al., 2003; Feder & Umali, 1993; Feder et al., 1985; Ghimire et al., 2015; Knowler & Bradshaw, 2007; Sunding & Zilberman, 2001; Tshikala et al., 2015; Yirga & Hassan, 2013; Zeng et al., 2018). The most common factors contributing to low technology adoption were absent or limited access to credit, information, and inputs, as well as inadequate infrastructure, social networks, and social learning behaviors. However, these studies do not address gender in technology adoption. The technology adoption decision often involves inputs from both men and women (Lambrecht et al., 2016; Marenya et al., 2015).

Many previous studies on gender and agricultural technology adoption note that women are less likely to adopt improved technologies compared to men (e.g., Chirwa, 2005; Diiro et al., 2015, 2018; Doss & Morris, 2001; Gaya et al., 2017; Kassa et al., 2013; Ragasa et al., 2012; Tiruneh et al., 2001). These studies found no difference between men and women in their adoption of agricultural technologies when controlled for other factors, such as complementary inputs. Yet, as Doss (2015) argued, in reality men and women do not have equal access to the inputs for which the model controls, such as capital, credit, land, seed, fertilizer, and extension services. Hence, it is important for policy planners on technology adoption to consider the differential levels of resource accessibility by gender. Manfre et al. (2018) also note that agricultural technologies are not gender neutral, and their adoption could be considerably influenced by social and cultural forces that affect existing gender roles and relations. This implies that gendered adoption of agricultural technologies varies widely based on context and culture (Gebre et al., 2019).

One of the most seminal studies on the relationship between gender and improved maize technology adoption is by Doss and Morris (2001). It focused on the gender of a farmer and the gender of the household head in Ghana. However, they did not provide information on *de jure* female-headed households and *de facto* female-headed households. This approach may mis-categorize married women with missing husbands as if they were living together in an MHH. Hence, it is important to note that *de facto* female-headed households and *de jure* female-headed households are not equally constrained to adopt improved maize technology^1^. Indeed, FAO (2010) notes that, while the *de facto* FHHs who receive remittance from their husbands can mitigate the effects of the absence of male agricultural laborers by increasing investment in farm tools and improved inputs, remittance does not procure labor itself. Besides, Doss and Morris’s (2001) analysis focused on the rate of technology adoption, not on the intensity of maize technology used. These assumptions made it difficult for the study to address gender inequalities regarding the adoption of improved maize technologies. Other seminal studies on gender and maize technology adoption include one by Seymour et al. (2016) in Ethiopia, Kenya, and Tanzania and one by Gebre et al. (2019) in Ethiopia. These studies focused on the adoption of improved maize varieties (IMVs); however, the maize technologies provided to farmers in a package are only complementary to each other in Ethiopia. Therefore, a decision to adopt one technology necessarily entails the decision whether to adopt another technology; hence varied outcomes are incremental at a societal level. Thus, research on the adoption of IMVs alone is not sufficient to formulate policy to enhance improved productivity.

Measuring gender differences in agricultural technology adoption typically takes either the gender of the individual farmer or household head as the unit of analysis. Male or female roles in technology adoption vary across countries and regions. In some regions of the Sahel, some plots are farmed individually by men or women, while others are farmed by household members (Doss, 2015). However, women’s access to key resources is commonly determined by their relationship with their male counterparts. In the Dawuro zone of southwestern Ethiopia, women in MHHs tend not to have separate farmland, while women in FHHs have farmland inherited from their husbands. Men have inherited property rights to land and other assets in the household, while women are normally supposed to be household heads only in the absence of their male counterparts. The household head owns the plots collectively farmed by household members (Gebre et al., 2021a). Thus, the present study used the gender of the household head as the unit of observation for data analysis.

This study investigates gender differences in the adoption rate and intensity of IMVs and chemical fertilizer in southwestern Ethiopia using data collected from Dawuro zone in 2018. The study uses a double-hurdle model to analyze the factors that affect the rate and intensity of maize technology adoption. The analysis classifies households as male-headed, female-headed, *de facto* female-headed (i.e., married women whose husband is absent), and *de jure* female-headed (i.e., widowed, divorced, or never married woman). Doing so, this study seeks to answer the following research questions: (1) Are there any significant gender differences in improved maize technology adoption and its intensity between male- and female-headed households? (2) What are factors affecting improved maize technology adoption and its intensity between male- and female-headed households?

Given the important role of women and associated household dynamics in Ethiopia, this research provides valuable insights into the food security status and overall well-being of the rural household in the nation by addressing gender differences in the adoption of maize technologies. We focus on maize because it takes the highest share (389.40 kcal) of national per capita calorie intake in Ethiopia, accounting for around 17–20% (Abate et al., 2015). Maize dominates rural consumption baskets, with 436 per capita calories, compared to only 107 per capita calories in urban areas. That is, per capita calorie consumption of maize in rural areas is four times that of urban areas in Ethiopia. The unit cost of calories from maize is the cheapest among all major cereals in Ethiopia, making it the most important cereal crop, particularly for economically less endowed households (Berhane et al., 2011; FAO, 2015; Rashid et al., 2010). Thus, maize is instrumental for the food security of Ethiopian households. Closing gender gaps in maize technology adoption would help improve maize productivity and associated food security of the farm households in the study area (Gebre et al. 2021b).

The rest of the article is organized as follows: section two discusses the general features of IMVs in Ethiopia, section three discusses the study area, data, and sampling, section four presents theoretical and empirical frameworks, section five presents results and discussion, and section six concludes the study.

## IMVs in Ethiopia

IMVs have two broad types: hybrids and open-pollinated varieties (OPVs). Hybrid varieties are not supposed to be recycled because of a drop in yield potential. Contrarily, OPVs can be recycled up to a maximum of three times without significantly losing yield potential. Hybrids have a higher yield potential than OPVs (Jaleta et al., 2013).

In 2013, at least 16 maize hybrids and 4 maize OPVs were under cultivation in Ethiopia, with hybrids accounting for 97% and OPVs 3% of the total maize seed market in Ethiopia. The Ethiopian seed market has been dominated by BH660, BH540, and pioneer hybrid maize varieties (Abate et al., 2015). From 2010 to 2013, the share of maize area covered by hybrids increased by 10%, while that of OPVs declined by 6% (Yirga et al., 2017). In Ethiopia, the improved maize seeds have been disseminated mainly via the Ethiopian Seed Enterprise, the major seed producer and distributor (Abate et al., 2015). According to the Ethiopian Seed Association (2014), regional public seed enterprises, private seed companies, cooperative unions, and out-grower farmers have recently been extensively involved in the production of hybrid maize seeds. Since most of these companies are grain producers or those shifted from other businesses, they tend to have limited knowledge and skills for hybrid maize seed production, which requires special technical knowledge and management skills.

## Study area, data and sampling

**Figure 1.**
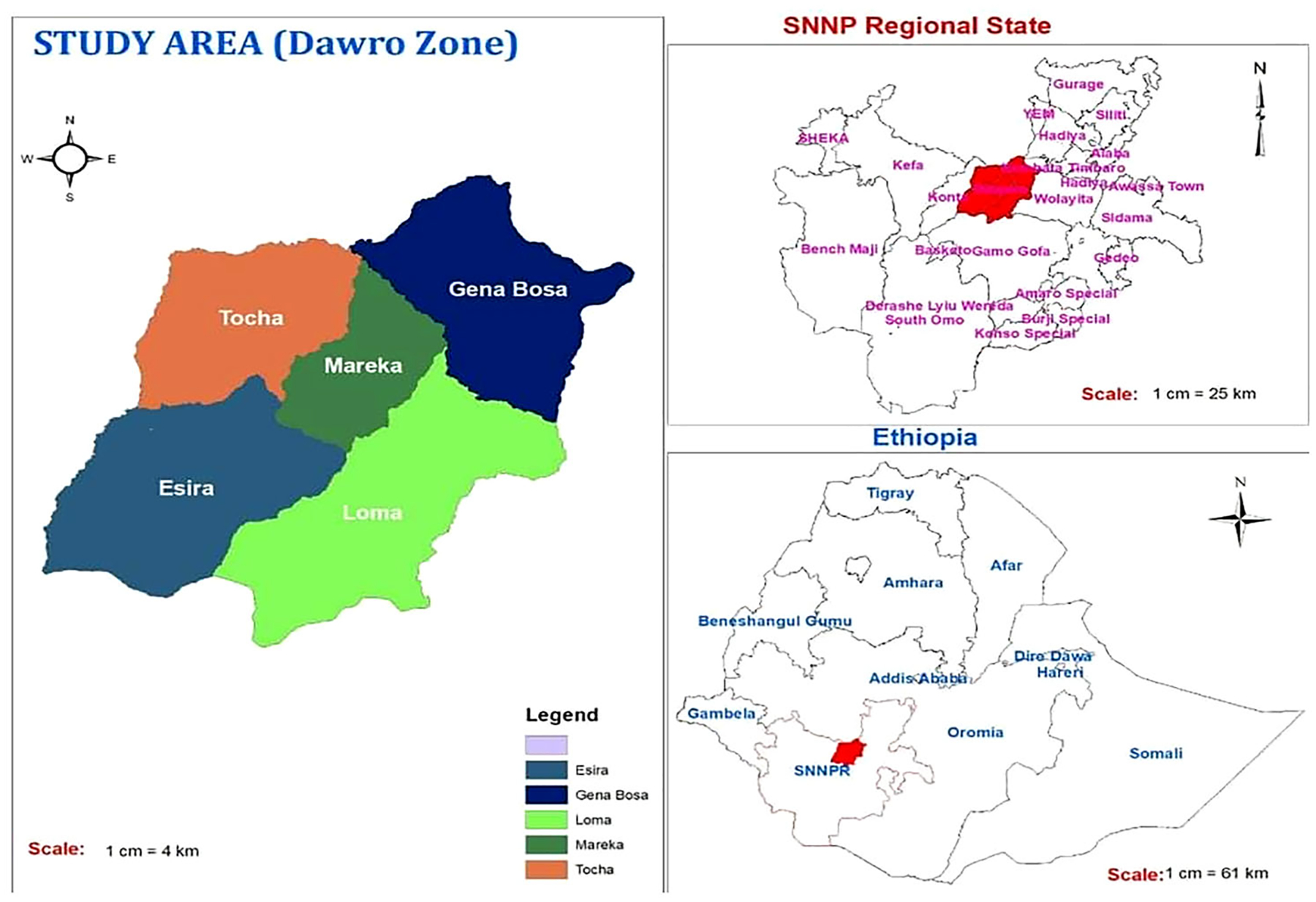
Map of the study area (Dawuro zone) in southwestern Ethiopia. Source: Authors’ creation.

This study is based on household and maize production data collected from the Dawuro zone of southwestern Ethiopia in 2018 (Figure 1). Three-stage purposive sampling techniques based on probability proportional to size were used to select districts, kebeles, and households. In the first stage, four districts, namely Loma (including Disa), Mareka, Tocha (including Tarcha zuria and Kachi) and Essara were selected based on their maize production potential. These districts are among the major maize growing areas in the country. In the second stage, 6–10 *kebeles* where maize was grown were selected from each district. In the third stage, on average, 20 maize-growing households were selected from each kebele for the survey, and finally, data was collected from 560 smallholder maize producers (409 MHHs and 151 FHHs). The selected 151 FHHs comprised 89 *de jure* and 62 *de facto* FHHs.

The collected information included details related to basic household composition, household membership for different social institutions, livestock ownership, off-farm incomes, inputs used for maize production, areas for maize cultivation and other socio-economic features of the household in the 2018 production season.

## Theoretical and empirical frameworks

### Theoretical framework

As noted in the extensive literature, the definition of “adopter” varies across studies (Doss, 2006). In this study, we defined an adopter as a farm household using either improved seed or chemical fertilizer, or both for maize production in 2017/18. We also consider adopter households as agents who make decisions in their best interests. Foster and Rosenzweig (2010) point out that the adoption of an input and its use is the outcome of optimizing by heterogeneous agents. This optimization takes place in the presence of budget constraints, asymmetric information, credit access, gender-specific factors, and the availability of technology and other inputs. Households are assumed to maximize their utility function subject to these constraints. Following similar assumptions and models adopted in a host of studies (Ali & Abdulai, 2010; Asfaw et al., 2012; Becerril & Abdulai, 2010; de Janvry et al., 2010; Kohansal & Firoozzare, 2013; Gebre et al., 2019), adoption decision can be modeled in a random utility framework.

In our model, the difference in the obtained utility between adoption *U*_*iA*_ and non-adoption *U*_*iN*_ of IMVs and chemical fertilizers is denoted as G*. Utility maximizing farm household, *i*, will choose to adopt either an IMV or chemical fertilizer, or both, if the utility gained from adopting is greater than the utility of non-adopting $\left(G_{i}^{*}=U_{iA}-U_{iN}>0\right)$ Since these utilities are unobservable, they can be expressed as a function of observable element in the following latent variable model:

(1) $$G_{i}^{*}=\beta X_{i}+u_{i}\text{ with }G_{i}=\{\begin{array}{ll} 1 & \text{if }G_{i}^{*}>1\\ 0 & \text{otherwise} \end{array}$$

where $G_{i}^{*}$ is a binary indicator variable that equals 1 if farm households growing an IMVs and/or use chemical fertilizer, and zero otherwise; *β* is a vector of parameters to be estimated; *X* is a vector of explanatory variables such as individual, household, and farm-level characteristics; and *u* is the error term.

### Empirical framework

The household adoption decision for IMVs and fertilizers is influenced by their decision of the proportion of the maize land planted to IMVs and the amount of fertilizer used per hectare (Chirwa, 2005; Doss & Morris, 2001). In our empirical model, we assume that adoption of IMVs and fertilizer involves two different decision stages: first, the rate of adoption (i.e., the proportion of households adopting IMVs only, fertilizer only, or both); and second, the intensity of adoption (i.e., the proportion of maize farmland devoted to the planting of IMVs and the amount of fertilizer applied per hectare). The dependent variable in the first stage is binary choice, and that in the second stage is continuous. Following Jones (1989), Gao et al. (1995), Newman et al. (2003), Langyintuo and Mungoma (2008), Ghimire et al. (2015), and Gebre et al. (2019), we employed a relatively new, innovative econometric tool—Cragg’s (1971) double-hurdle model—in data analysis. The double-hurdle model assumes that two separate hurdles must be crossed in order to report a positive intensity of technology use. Different sets of explanatory variables determine the two separate decisions. Since the *de facto* FHHs and *de jure* FHHs have fewer observations relative to MHHs, we combined them into a simple “FHHs” category for model analysis.

Agricultural technologies are interdependent, and farmers simultaneously adopt them as complements, substitutes, or supplements. In Ethiopia, improved seeds and chemical fertilizers are disseminated in a package for the introduction of new maize technology. Thus, maize farm households in Ethiopia will decide whether to adopt IMVs, fertilizer or both. Since IMVs and fertilizer are complementary to each other in a package, the benefits obtained when both technologies are adopted jointly exceed the benefits achieved when only either one is adopted, for the same number of times of adoption.

In our double-hurdle model, we assume that the decision to adopt IMVs can be affected by the decision to adopt chemical fertilizer or vice versa because the two adoption decisions can be linked. We applied a two-step probit approach for the first hurdle to determine the probability of the household adoption of either IMVs or fertilizer, or both. In the first step, a full set of estimators is incorporated into the maximum likelihood (ML) probit estimation to predict the probability of adopting either fertilizer or improved maize seeds. The parameters estimated from this step are not presented. In the second step, the predicted values for the respective adoption of IMVs and fertilizer are included in IMVs adopter and fertilizer adopter equations as independent variables in the final set of estimations. In this case, the two dependent variables are jointly determined; we just put each on the right-hand side of the other equation. According to Greene (2008), this model is a recursive, simultaneous equation for bivariate probit estimation. Because of the hypothesized endogeneity of the system, the two equations are estimated simultaneously (Chirwa, 2005; Doss & Morris, 2001).

The truncated regression model was used for the second hurdle to determine the intensity of maize technology adoption. The truncated regression is proposed for a dependent variable for which its distribution is not representative of the entire population. We performed truncated regression in the way that all the zero values from the bivariate probit model (for those who adopted neither IMVs nor fertilizers) were truncated, and that only positive values (regarding the proportion of maize land allocated to IMVs and the amount of fertilizer used per hectare) were included in the regression model. The truncated regression model was created using the same explanatory variables for both dependent variables. This model uses ML to estimate the effect of the changes in explanatory variables on the expected value of the dependent variable given a normal distribution.

Both hurdles are assumed to be linear in their parameters (*β*, *α*), with additive disturbance terms ε and υ normally distributed. The double-hurdle model is specified as follows:

(2) $$y_{1}^{*}=\beta_{1}X_{1}+\beta_{2}\text{ Fertadopter}+\varepsilon_{1},y_{1}=1\text{ if }y^{*}>0,\;0\text{ otherwise, decision to adopt an IMVs }$$

(3) $$y_{2}^{*}=\beta_{3}X_{2}+\beta_{4}\text{ IMVadopter}+\varepsilon_{2},\;y_{2}=1\text{ if }y^{*}>0,\;0\text{ otherwise, decision to adopt fertlizer }$$

(4) $$Z_{1}^{*}=\alpha_{1}M_{1}+\upsilon_{1},\;Z_{1}=1\text{ if }Z^{*}>0,\;0\text{ otherwise, intensity of IMVs adoption }$$

(5) $$Z_{2}^{*}=\alpha_{2}M_{2}+\upsilon_{2},\;Z_{2}=1\text{ if }Z^{*}>0,\;0\text{ otherwise, intensity of fertilizer adoption }$$

where, y* is a latent variable that represents the probability of the household’s decision to adopt IMVs and/or fertilizer, and z* is a latent variable representing the intensity of adoption (proportion of maize land planted to IMVs and the amount of fertilizer applied per hectare); X and M are explanatory variables for adoption decision and use intensity, respectively; the variables denoted as “Fertadopter” and “IMVadopter” will show predicted values (generated from first step probit estimation) that are expected to solve the endogeneity problem in Equations (2) and (3); α and *β* are the parameters to be estimated, and ε and υ are the respective error terms assumed to be independent and normally distributed as ε_*i*_=*N*(0, 1) and υ_*i*_=*N*(0, α^2^). To assess the effect of the explanatory variables on the rate of maize technology adoption, the marginal effects after the bivariate probit model were estimated.

In addition to the various factors that are expected to influence the rate of the adoption of IMVs and/or fertilizer, we assume that certain technology-specific factors influence the two adoption decisions separately. In the case of IMVs, varietal choice, and in the case of fertilizer, soil fertility conditions, are expected to influence adoption decisions, respectively. In the study area, the choice of an IMV is decided by the local agriculture office in consideration of the local agro-ecology before the seeds are supplied to farmers. Therefore, in the fertilizer adoption equation, we included the soil fertility variable in relation to the crop rotation practices adopted on the maize farmland.

## Results and discussion

### Descriptive statistics

Table 1 shows the summary statistics regarding the variables of our interest. In order to examine gender differences in maize technology adoption, we divided sampled households into four groups: MHHs, *de facto* FHHs, *de jure* FHHs, and FHHs (*de facto* FHHs and *de jure* FHHs combined). On average, 64.8% and 65.5% of sampled households used IMVs and chemical fertilizers, respectively; 55.4% of maize land was planted with IMV seeds, while 57.7 kg of fertilizer was applied per hectare. Compared to MHHs, a significantly lower proportion of FHHs adopted IMVs and fertilizer. Among FHHs, a lower proportion of *de jure* FHHs adopted them than *de facto* FHHs. The proportion of maize farmland planted with IMVs and the amount of fertilizer used was significantly greater in plots managed by MHHs than those by FHHs (*p* < 0.10). This result is similar to Ragasa et al. (2012). The types of IMVs used by sampled households were BH540, BH660/1, and Pioneer. Their adoption was related to the local agro-ecological conditions. Urea, DAP (di-ammonium-phosphate), and NPSB (Nitrogen-Phosphorous-Sulfur-Born) were the major fertilizers used by households.

The average age of the head of the sampled households was 42.61 years with a higher average in FHHs. Compared to *de facto* FHHs; *de jure* FHHs are older in age. This could be linked to the migration of young married men in search of better income elsewhere. For rural households that are unable to hire labor from the market, labor availability depends on the amount of family labor. The result of this study shows that the number of male labor is significantly higher for MHHs (*p* < 0.01), while female labor is higher for FHHs. This result is consistent with the results of Djurfeldt et al. (2018) regarding six African countries.

The average number of livestock (measured in Tropical Livestock Units) owned by the sample households was 6.03 TLU. MHHs own an average of 6.43 TLU, whereas FHHs own an average of 4.95 TLU, with a significant difference (p < 0.01). This implies that an average MHH has a higher asset holding status than an average FHH. Among FHHs, *de facto* FHHs own more livestock than *de jure* FHHs. This indicates that the *de facto* FHHs had a higher asset holding status than *de jure* FHHs. This result is consistent with other studies (i.e., FAO, 2011). Additionally, the average number of oxen owned by MHHs was higher than that of FHHs. This result is also in line with FAO (2011), which notes that “women own fewer of the working animal needed in farming” (pp. 15). As for the ownership of oxen among FHHs, the *de facto* FHHs have more oxen than the *de jure* FHHs, which is likely related to their higher access to financial resources associated with remittances from and credit through their husband. The superior ownership of oxen by the *de facto* FHHs implies that they are more likely to use animal traction.

**Figure 2.**
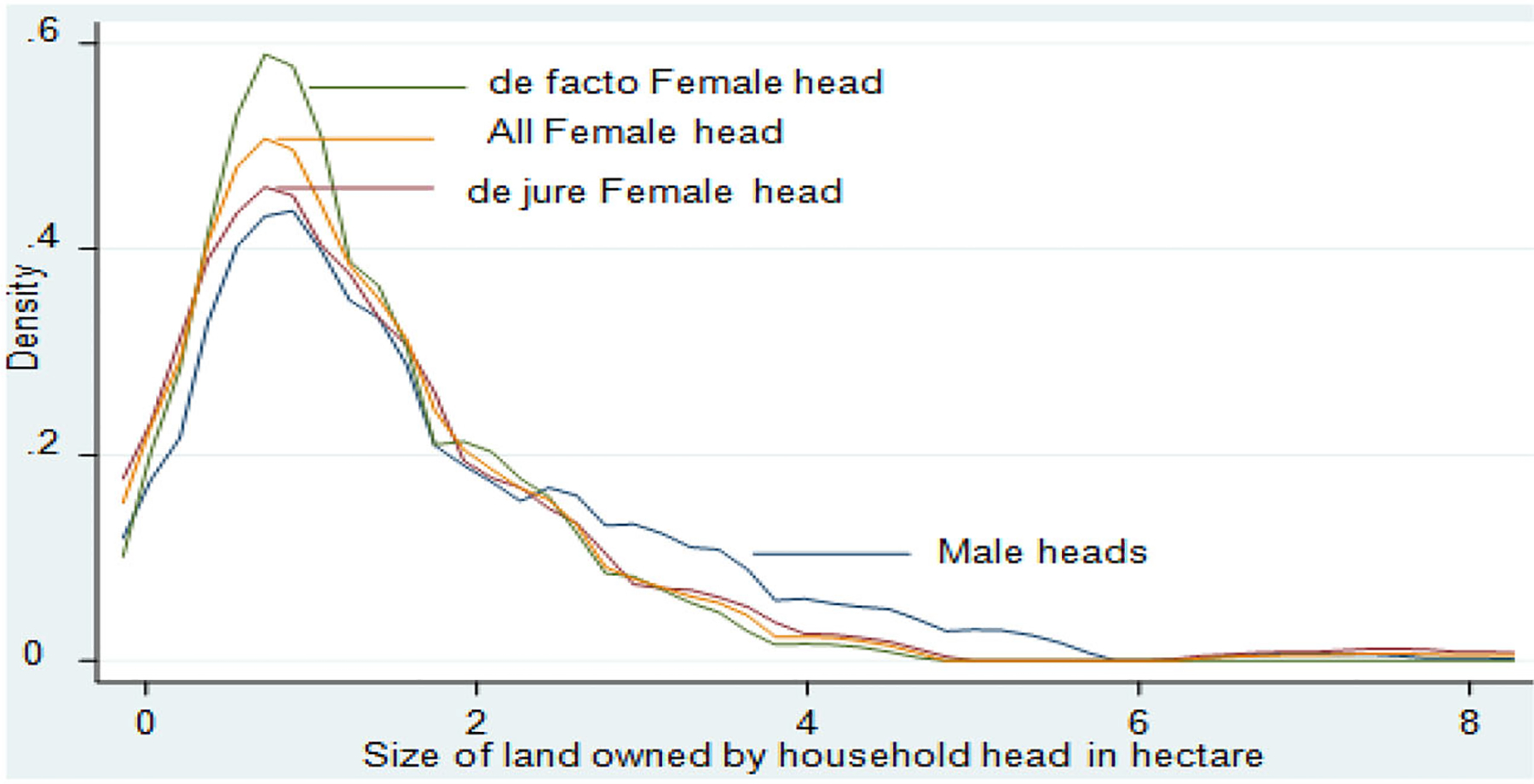
Distribution of landholding between MHHs and FHHs, de facto FHHs, and de jure FHHs — Kernel Density estimation. Source: Author’s computation from the survey data (2018).

The total average landholding for sampled households was 1.58 ha, which is higher than the national and regional average holdings of 1.02 ha and 1.23 ha, respectively. The average land held by MHHs was significantly higher than that held by FHHs, which is in line with previous studies (i.e., Agarwal, 2015; FAO, 2011). Regarding the landholding status among FHHs, the average land owned by the *de facto* FHHs is less than the *de jure* FHHs. This would suggest that some or many adult male partners of rural households leave home to seek nonfarm job opportunities elsewhere because of a shortage of their own farmland for sustaining their livelihood. This result is in contrast with the result of Djurfeldt et al. (2018). The gender difference in landholding becomes more apparent when we compare the landholding distribution between MHHs and FHHs (Figure 2). The distribution of landholding for FHHs is predominantly at the left of the MHHs distribution. The landholding distribution for *de facto* FHHs and *de jure* FHHs nearly overlap except the lower middle tail. The average land devoted to maize cultivation in the 2017 production season by MHHs and FHHs was 0.90 and 0.60 ha, respectively. However, among FHHs, *de jure* FHHs use more land area to cultivate maize. The key reason for this could be related to their larger landholdings compared to *de facto* FHHs.

Households were also asked whether they accessed credit services during the maize production period. About 47% of MHHs and 36% of FHHs have access to credit from financial institutions. Among female heads, 39% of *de facto* FHHs and 33% of *de jure* FHHs have access to credit services. This implies that *de facto* FHHs still benefit from their husbands’ names and social network to access financial services in the community. Extension services such as visits by and advice received from agricultural experts are designed to improve the farm productivity of rural households. However, evidence (e.g., FAO, 2011; Ragasa et al., 2012) suggests that female heads tend to lag behind men in exploiting the benefits from extension services. The result of this study shows that approximately 78% of the sampled households have access to extension agents. Meanwhile, FHHs have less access to extension agents than MHHs. Among FHHs, *de facto* FHHs have more access to extension agents than *de jure* FHHs. This implies that an extension agent is more likely to visit *de facto* FHHs than *de jure* FHHs. The main reason for more access to extension services by *de facto* FHHs might be related to the name of the husband and his social networks.

### Empirical results

Tables 2 and 3 show the results of the double-hurdle models. In both hurdles, the gender variable lacks significant explanatory power. As a further test of the significance of gender on adoption, a Wald-test statistics was constructed to test the hypothesis that the coefficient of the gender of the household head is equal to 0 (*H*_0_: *I*(Gender) = 0). The result showed that for both hurdles, the null hypothesis failed to be rejected. This suggests that controlling for other factors, neither IMVs adoption nor fertilizer adoption is associated with the gender of the household head. That is, the gender difference in maize technology adoption is associated with the gender-differentiated division of labor and access to complementary inputs. Other studies have reported similar results (e.g., Chirwa, 2005; Doss & Morris, 2001; Ragasa et al., 2012).

### Factors affecting adoption decision

Table 2 presents the results from the first hurdle analysis regarding the probability of the adoption of IMVs and fertilizer by the gender of the household head. Of the variables included in the model, the number of adult male labor, the number of female labor, the size of farmland, extension contact, access to credit, training, market information, off-farm income, participation in FTC, and the adoption of crop rotation were found to affect the adoption of IMVs or fertilizer. Regarding the pooled household sample, for both IMVs and fertilizer, the probability of adoption was positively influenced by the number of adult males but negatively influenced by the number of adult females in the household. The negative relationship with the number of adult females indicates that the greater the number of female members in the household, the less likely it is to adopt either an IMV or fertilizer. This result might be related to the gendered division of labor in the households in Dawuro zone where men tend to prepare the land and plant maize. In this area, it is considered culturally inappropriate for women to plow land with oxen for crop production. The positive effect of adult male labor in the household is consistent with previous studies (e.g., Doss & Morris, 2001).

In the FHHs, the probability of adopting IMVs was positively influenced by the number of adult males, but negatively influenced by the number of adult females. The probability of fertilizer adoption was positively influenced by the number of males for both MHHs and FHHs. Our possible explanation for this result is that chemical fertilizer is a relatively complex technology, in the sense that a household head who decides to adopt fertilizer must have knowledge about the proper rate and composition of application. Thus, a male farmer who is experienced in farming positively influences the probability of fertilizer adoption. A girl or woman can learn how to use fertilizer if she is guided for such knowledge. There may be some cultural barriers to women that prohibit them from using chemical fertilizer; this would be related to the general gendered division of labor for agriculture in Dawuro zone where men tend to prepare land and plant maize.

The farmland size was found to positively influence the probability of both IMVs and fertilizer adoption of any household type. This finding is consistent with the findings of Doss and Morris (2001) and Ghimire et al. (2015). Our finding suggests that households with a smaller land for maize cultivation consider it economically not very efficient to use either IMVs or fertilizers. Since land ownership is an indicator of wealth, an increase in land size increases the likelihood of adopting IMVs and fertilizer. Credit was found to significantly influence the probability of IMVs and fertilizer adoption for both MHHs and FHHs, implying that households tend to utilize credit loans for the purchase of agricultural inputs, such as IMVs and chemical fertilizer. This result is in contrast to Asfaw et al. (2012). In addition, extension contact, market information and participation in farmer training positively and significantly influenced the adoption rates of IMVs and fertilizer for both MHHs and FHHs, which is consistent with the finding of previous studies (Asfaw et al., 2012; Ghimire et al., 2015; Ragasa et al., 2012). Their significantly positive influence indicates the significant efficiency of the agricultural and marketing extension system for maize technology adoption in southern Ethiopia.

For MHHs, we found negative association between off-farm income and probability of IMVs and fertilizer adoption, which is in contrast to the findings of Langyintuo and Mungoma (2008). Our finding may imply that, as household heads access more off-farm income, they tend to shift their investment from maize farming technologies to other income sources. However, off-farm income has no significant effect on adoption decisions for FHHs.

### Intensity of adoption

In this section, we present the results of the intensity of IMVs and fertilizer adoption using the second step double-hurdle model. We performed truncated regression in this step in which only positive values obtained from the first hurdle analysis are reported. Table 3 shows that the number of adult male, land size, oxen ownership, market information and access to credit significantly influenced the amount of maize farmland allocated to IMVs and the amount of fertilizer applied in hectare for both MHHs and FHHs. The positive relationship between the number of adult males and the intensity of IMVs and fertilizer adoption indicates that an additional number of adult male laborers in the household increases the amount of land allocated to IMVs and fertilizer application rate. The significant and positive relationship between land size and intensity of adoption suggests that any additional maize farmland was devoted to the planting of IMVs and chemical fertilizer application for both MHH and FHHs. This finding is contrary to the findings of Ghimire et al. (2015). The positive relationship between the ownership of oxen and intensity of adoption implies that an additional number of oxen owned increases the amount of land allocated to IMVs planting and fertilizer application for both MHHs and FHHs. This finding is consistent with Asfaw et al. (2012).

Access to credit was found to be negatively associated with the intensity of IMVs planting and fertilizer application. This suggests that credit access has a heterogeneous effect on technology adoption and its use; it has a positive influence on growers’ adoption of IMVs and fertilizer, but negative effect on their use. One explanation could be that maize farmer households utilized credit for purchase of agricultural inputs including IMVs and fertilizer, whereas any additional loan was diverted for the purchase of other on-farm inputs or nonfarm income generating activities.

Participation in farmer training was found to positively affect the intensity of IMVs planting for MHHs and fertilizer application for both MHHs and FHHs, with statistical significance. This implies that the more training farmers participate in, the more intensity of IMVs planting and fertilizer application farm households would have, hence highlighting the importance of training in improving farmers’ ability to manage and use new technologies. This finding is similar to Ghimire et al. (2015), but contrasts with the finding of Ragasa et al. (2012).

## Conclusion

This study has examined gender differential in maize technology adoption by analyzing the key factors influencing the adoption of IMVs and fertilizer and intensity of their adoption using sample data of farm households in Dawuro zone, Ethiopia. The two-tiered analysis of adoption and its intensity drew on the use of a double-hurdle model. Gender differences on the basis of the two household categories, MHHs and FHHs, were examined using a simple independent t-test. The descriptive result showed that there was a difference among the three household types in the rate and intensity of maize technology adoption. Among them, the *de jure* FHHs were the most disadvantaged in both IMVs and fertilizer adoption. Additionally, MHHs had more productive resources than FHHs. The *de jure* FHHs were the most disadvantaged in this regard. The simple mean comparison test suggested a significant difference in maize technology adoption between MHHs and FHHs.

However, after controlling for other factors, the gender of the household head did not significantly affect the maize technology adoption in the study area. This indicates that the difference in adoption was derived from gender-differentiated access to productive resources rather than the biological difference of the household heads. The factors such as land size, oxen ownership, male labor, extension contact, training, credit, and market information positively influenced the adoption decision of both MHHs and FHHs; however, the degree of influence was less for FHHs. Off-farm income and female labor negatively affected maize technology adoption in the study area. Since technology adoption is a process, credit had a heterogeneous effect over these processes. We recommend increasing the access of FHHs to productive resources, particularly *de jure* FHHs in Ethiopia, to enable them to adopt maize farming technology in order to improve their wellbeing.

## Figures and Tables

**Table 1.** Descriptive statistics and results from test and mean differences by gender of household head.

Variable		Pooled sample	MHHs	All FHHs	de facto FHHs	de jure FHHs	Difference MHH vs. FHH
Number of observations		560	409	151	62	89	
Outcome variable							
IMVs adopter		0.648	0.6490	0.6479	0.6935	0.6292	0.0010
Chemical fertilizer adopter		0.655	0.6556	0.6550	0.6774	0.6401	0.0006
Proportion of maize land planted with IMVs		0.554	0.5647	0.5264	0.5772	0.4536	0.0383*
Amount of fertilizer applied (kg/ha)		0.577	0.561	0.5091	0.5435	0.4596	0.0520*
Independent variables							
Gender of head		1.00	0.730	0.270	0.111	0.159	0.460***
Size of the household		6.185	6.660	4.900	5.322	4.673	1.759***
Number of children in the household (≤15 years)		2.119	2.398	1.364	1.435	1.284	1.034***
Number of males in the household (≥15 years)		2.185	2.381	1.655	1.767	1.442	0.725***
Number of females in the household (≥15 years)		1.876	1.867	1.900	1.967	1.957	−0.032
Age of the household head in years		42.612	42.202	43.721	41.193	45.389	−0.138***
Education level of the household head in years		3.434	3.477	3.317	3.548	3.157	0.160*
Number of livestock in TLU		6.033	6.435	4.946	6.503	4.737	1.488***
Number of oxen owned by household		1.548	1.665	1.231	1.370	1.242	0.433***
Total landholding in hectares		1.583	1.708	1.245	1.238	1.395	0.463***
Size of land planted to maize in hectares		0.820	0.899	0.605	0.653	0.700	0.294***
Access to credit service		0.382	0.473	0 .361	0.389	0.334	0.112**
Contact with an extension agent		0.780	0.799	0.728	0.790	0.7368	0.071*
Participation in social activities		0.6589	0.7096	0.6528	0.6754	0.6516	0.0568*
Participation in farmer training center (FTC)		0.7232	0.7512	0.7284	0.7640	0.6774	0.0228
Distance from farmer training center in km		2.112	2.040	2.307	2.1766	2.3994	−0.2669*
Access to market information		0.7107	0.7188	0.6887	0 .7640	0.5806	0.0300
Access to off-farm income		0.0767	0.0806	0.0662	0.0967	0.0449	0.0144
Crop rotation at maize farmland		0.5910	0.5941	0.5727	0.5967	0.5730	0.0214
IMVs used	BH540	0.7720	0.7744	0.7653	0.7142	0.8035	0.0091
	BH660/1	0.0989	0.1015	0.0918	0.0952	0.0893	0.0097
	Pioneer	0.1291	0.1240	0.1428	0.1904	0.1071	−0.1377
Chemical fertilizer used	Urea	0.1159	0.0922	0.1800	0.0930	0.2456	−0.0879
	DAP	0.8436	0.8671	0.7800	0.8837	0.7017	0.0871
	NPSB	0.0403	0.0405	0.0400	0.0232	0.0526	0.0005
Agro-ecology	Low land	0.566	0.572	0.549	0.584	0.50	−0.006
	Mid-land	0.384	0.386	0.377	0.348	0.419	−0.006
	High land	0.05	0.041	0.072	0 .067	0.080	0.009

Source: Survey result (2018).

***, ** and * denote level of significance at 1%, 5% and 10%, respectively.

**Table 2.** Growers’ adoption of IMVs and fertilizer by gender of HH head.

Variable	Pooled Sample	MHHs	FHHs
IMVs adoption			
Gender of head (1 = Female)	−0.0244 (0.1513)		
Number of children	0.0238 (0.0451)	0.0110 (0.0538)	0.0589 (0.0963)
Number of adult males	0.1265*** (0.0586)	0.0830 (0.0695)	0.2387* (0.1271)
Number of adult females	−0.1247** (0.0642)	−0.0617 (0.0804)	−0.2675** (0.1302)
Age of head	0.0012 (0.0074)	0.0027 (0.0090)	−0.0055 (0.0176)
Education of head in years	0.1321 (0.0914)	0.1344 (0.1158)	0.1769 (0.1722)
Landholding in hectare	0.0998** (0.0476)	0.1413** (0.0629)	0.0071* (0.0717)
Livestock in TLU (except oxen)	0.0866 (0.1275)	0.0779 (0.1542)	0.0449 (0.2513)
Ownership of oxen	0.0837 (0.0904)	0.1621 (0.1181)	−0.0064 (0.1662)
Access to credit	0.4904** (0.2045)	0.5668** (0.2644)	0.1914* (0.3598)
Participation in FTC	0.7593*** (0.2013)	0.6965*** (0.2454)	1.0551*** (0.3965)
Extension contact	0.7440*** (0.2357)	0.8629*** (0.2833)	0.7986** (0.4997)
Access to market information	0.8443** (0.1687)	0.8717*** (0.2008)	0.9543** (0.3986)
Off-farm income	−0.6227*** (0.2464)	−1.0028*** (0.2969)	0.3722 (0.6247)
Participation in social events	0.1331 (0.1458)	0.1440 (0.1732)	0.0949 (0.3231)
Fertilizer predictor	0.1338 (0.0884)	0.1412* (0.0832)	−0.0265 (0.2892)
Constant	−1.9940*** (0.5070)	−2.2774*** (0.5994)	−1.8586** (0.7624)
Wald test statistic: *β* Female = 0	0.7892		
Fertilizer adoption			
Gender of head (1 = Female)	−0.0301 (0.1529)		
Number of children	0.0501 0.0500)	0.0341 (0.0552)	0.1214 (0.1122)
Number of adult males	0.1703*** (0.0609)	0.1281* (0.0773)	0.2922** (0.1758)
Number of adult females	−0.1060** (0.0659)	−0.0459 (0.0833)	−0.25944 (0.1639)
Age of head	0.0020 (0.0076)	0.0056 (0.0104)	−0.0137 (0.0222)
Education of head in years	0.1230 (0.0997)	0.1380 (0.1332)	0.0220 (0.2827)
Landholding in hectare	0.1154** (0.0513)	0.1408** (0.0630)	0.0078* (0.0819)
Livestock in TLU (except oxen)	0.1134 (0.1338)	0.1311 (0.1575)	0.0714 (0.2814)
Ownership of oxen	0.0378 (0.0912)	0.0996 (0.1150)	0.0476 (0.2101)
Access to credit	0.5088** (0.2116)	0.5338* (0.2788)	0.2725* (0.6516)
Participation in FTC	0.7536*** (0.2037)	0.6798*** (0.2524)	0.9705*** (0.3699)
Extension contact	0.8278*** (0.2319)	0.9487*** (0.2991)	0.7118** (0.4138)
Access to market information	0.9297*** (0.1805)	0.9754*** (0.1992)	0.8112** (0.6634)
Off-farm income	−0.5050*** (0.2578)	−0.8941*** (0.3104)	0.4911 (0.7016)
Participation in social events	0.1456 (0.1474)	0.1534 (0.1982)	0.1483 (0.3852)
Crop rotation	0.1401* (0.0848)	0.1640 (0.1524)	0.1052 (0.3336)
IMVs predictor	0.0908 (0.0771)	0.0872 (0.0989)	0.3121 (0.5443)
Constant	−2.4056*** (0.4595)	−2.8153*** (0.6709)	−1.4203** (0.8873)
Log likelihood	−239.6030	−168.7014	−62.3467
Wald (*X*^2^) test : *β* Gender = 0	0.9382		
Observation	560	409	151

Source: Survey result (2018).

***, ** and denote level of significance at 1%, 5% and 10%, respectively. Standard Errors are given in parentheses.

**Table 3.** Growers’ adoption intensity of IMVs and fertilizer by gender of HH head.

Variables	Pooled Sample	MHHs	FHHs
IMVs adoption intensity			
Gender	−0.0032		
Number of children	0.0079	0.0041	0.0175
Number of adult males	0.0317**	0.0208	0.0557
Number of adult females	−0.0268*	−0.0116	−0.0572***
Age of head	0.0003	0.0008	−0.0018**
Education of head in years	0.0276	0.0283	0.0263
Landholding in hectare	0.0233***	0.0294***	0.0012
Livestock in TLU (except oxen)	0.0206	0.0204	0.0117**
Ownership of oxen	0.0157	0.0289	0.0027
Access to credit	0.1084**	0.1156**	0.0476**
Participation in FTC	0.1633***	0.1439**	0.2215***
Extension contact	0.1705***	0.1866***	0.1659**
Access to market information	0.1902**	0.1898**:	0.0895**
Off-farm income	−0.1288**	−0.2007**	0.1952
Participation in social events	0.0318	0.0307	0.0246
Fertilizer predictor	0.0164	0.0185	−0.0037
Fertilizer adoption intensity			
Gender	−0.0058		
Number of children	0.0073	0.0044	0.0175
Number of adult males	0.0313***	0.0208***	0.0557***
Number of adult females	−0.0259	−0.0116	−0.0572
Age of head	0.0003	0.0008	−0.0018
Education of head in years	0.0284	0.0283	0.0263
Landholding in hectare	0.0232**	0.0294***	0.0016*
Livestock in TLU (except oxen)	0.0212	0.0203	0.0117
Ownership of oxen	0. 0147	0.0289	0.0027
Access to credit	0.1094***	0.1156***	0.0476**
Participation in FTC	0.1666***	0.1439***	0.2215***
Extension contact	0.1701**	0.1866**	0.1659**
Access to market information	0.1925*	0.1898**	0.1952
Off-farm income	−0.1278***	−0.2007***	0.0895**
Participation in social events	0.0302	0.0307	0.0246
Crop rotation practice	0.0110**	0.0127*	0.0080
IMV predictor	0.0072	0.0067	0.0239

Source: Survey result, (2018).

***, ** and * denote level of significance at 1%, 5% and 10%, respectively.

## Data Availability

Authors do not have the right to share the data. However, it may be made available to the reader upon request.

## Footnotes

1. In this study, *de jure* FHHs are defined as households headed by women who are not married, are divorced or widowed while *de facto* FHHs are defined as households headed by women whose spouses (husbands) are away from home because of work or other reasons.
